# LncRNAs divide and rule: The master regulators of phase separation

**DOI:** 10.3389/fgene.2022.930792

**Published:** 2022-08-10

**Authors:** Kumaravel Somasundaram, Bhavana Gupta, Nishkarsh Jain, Samarjit Jana

**Affiliations:** Department of Microbiology and Cell Biology, Indian Institute of Science, Bangalore, India

**Keywords:** lncRNA, phase separation, biomolecular condensates, multivalency, intrinsically disordered region, N6-methylAdenosine (m6A), RNA granules, RNA binding proteins

## Abstract

Most of the human genome, except for a small region that transcribes protein-coding RNAs, was considered junk. With the advent of RNA sequencing technology, we know that much of the genome codes for RNAs with no protein-coding potential. Long non-coding RNAs (lncRNAs) that form a significant proportion are dynamically expressed and play diverse roles in physiological and pathological processes. Precise spatiotemporal control of their expression is essential to carry out various biochemical reactions inside the cell. Intracellular organelles with membrane-bound compartments are known for creating an independent internal environment for carrying out specific functions. The formation of membrane-free ribonucleoprotein condensates resulting in intracellular compartments is documented in recent times to execute specialized tasks such as DNA replication and repair, chromatin remodeling, transcription, and mRNA splicing. These liquid compartments, called membrane-less organelles (MLOs), are formed by liquid–liquid phase separation (LLPS), selectively partitioning a specific set of macromolecules from others. While RNA binding proteins (RBPs) with low complexity regions (LCRs) appear to play an essential role in this process, the role of RNAs is not well-understood. It appears that short nonspecific RNAs keep the RBPs in a soluble state, while longer RNAs with unique secondary structures promote LLPS formation by specifically binding to RBPs. This review will update the current understanding of phase separation, physio-chemical nature and composition of condensates, regulation of phase separation, the role of lncRNA in the phase separation process, and the relevance to cancer development and progression.

## Introduction

Human genome sequencing came up with a massive surprise that only less than 2% of the genome is translated and the remaining ∼98% does not encode a protein, creating loopholes in the central dogma. This non-coding part of the genome was considered “junk or dark matter,” with the exceptions of rRNAs, tRNAs, snRNAs, and snoRNAs (Lander et al., 2001). It was unknown that this junk would belong to a new and larger class of transcripts called lncRNAs (long non-coding RNAs). This class represents transcripts of more than 200 nucleotides in length, lacking coding potential; however, some can encode small peptides (Zhang et al., 2018). LncRNAs have unique biochemical properties such as their interaction with DNA, RNA, and protein and their ability to fold into intricate secondary structures that allow them to interact with multiple RBPs (RNA-binding proteins). After the discoveries of gene-regulatory lncRNAs H19 (Brannan et al., 1990) and Xist (X-inactive specific transcript) (Brown et al., 1991) in the 90s, the number of reports of functionally important lncRNAs has increased continuously. From the nucleus to the cytoplasm, they have been linked to the regulation of several cellular processes such as DNA replication and repair, chromatin remodeling, transcription, mRNA splicing, translation, turnover, and signaling pathways. Over the past 2 decades, extensive research has resolved various queries related to lncRNAs. Moreover, a new concept of LLPS (liquid–liquid phase separation) is coming to light, which is believed to answer countless unanswered questions about the cellular world.

Moreover, a bridge between LLPS and lncRNA has been formed, giving a new meaning to the subcellular localization of lncRNA, where it can perform its functions. It has been shown that phase condensates have a dynamic character, and this property can give lncRNAs the ability to perform their regulatory roles rapidly and transiently. Several functional lncRNAs have been known for years, but what makes them unique if they participate in phase condensation will be discussed in this review. It will also be discussed how condensates aid the lncRNAs in playing a pivotal role in fine-tuning many physiological and pathological activities. The content below will also talk about the connection between LLPS and lncRNA in cancer.

## Phase separation

The presence of a lipid bilayer helps in compartmentalization and allows eukaryotic cells to organize biochemical reactions in space and time. But one fundamental question remains: how do cells maintain biomolecular interactions and homeostasis in the cytoplasm and nucleus. One way to coordinate spatiotemporal regulation is to control the localization of specific biomolecules at one place while excluding others, thereby creating a heterogeneous environment. Work from past years has shown the existence of such functional membrane-less compartments in the nucleus and cytoplasm. These compartments include the pericentriolar matrix which plays a role in microtubule nucleation (Woodruff et al., 2017), the nucleolus which produces and assembles ribosomes (Boisvert et al., 2007), Cajal bodies for assembling spliceosome machinery (Gall, 2003), and so on. Even though these compartments were discovered decades ago, the involvement of physio-chemical forces and biological contents driving the formation of these bodies is still unfolding. Research has shown that such compartments form through liquid–liquid phase separation (LLPS) or condensation. Because of diversity in their molecular composition, location, and function, “biomolecular condensates” is considered an umbrella term for these membrane-less cellular compartments that form because of condensation (Table 1, Banani et al., 2017; Shin and Brangwynne, 2017).

**TABLE 1:** List of known phase condensates.

S. No.	Location	Occurrence	Name	Function	References
1	Nucleus	Ubiquitous	Nucleolus	Ribosomal biogenesis	Boisvert et al. (2007)
2		Ubiquitous	Promyelocytic leukemia (PML) body/Kremer body/PML oncogenic domain	Regulates transcription, apoptosis and anti-viral defense	Lallemand-Breitenbach and de Thé (2010)
3		Ubiquitous	Cajal body	RNA processing and spliceosomal machinery assembly	Gall. (2003), Nizami et al. (2010), Handwerger et al. (2004)
4		Ubiquitous	Polycomb group (PcG) bodies	Repression of transcription	Pirrotta and Li (2012)
5		Ubiquitous	Nuclear speckle/Splicing factor (SF) compartment	RNA processing such as mRNA splicing	Spector and Lamond (2011)
6		Ubiquitous	Gems/Gemini of cajal bodies	Storage histone mRNA processing	Morimoto and Boerkoel, (2013), Cauchi, 2011
7		Ubiquitous	Cleavage bodies	mRNA processing	Li et al. (2006)
8		Ubiquitous	Histone locus bodies	Histone mRNA processing	Nizami et al. (2010), Duronio and Marzluff (2017)
9		Cell-type specific	Paraspeckle	Transcription regulation and storage of certain RNAs	Fox and Lamond (2010, Henning et al. (2015)
10		Condition-dependent	OPT domains/53P1-OPT domains	Transcription regulation	Spector (2001), Harrigan et al. (2011)
11		Condition-dependent	DNA damage foci	DNA damage pathway	Altmeyer et al. (2015)
12		Condition-dependent	Nuclear stress bodies	Transcriptional and splicing regulation	Biamonti and Vourc’h, (2010), Niemela et al. (2019)
13		Condition-dependent	Peri-nucleolar compartment	RNA metabolism, linked with malignancy	Pollock and Huang (2010), Norton and Huang (2013)
14		Condition-dependent	Amyloid bodies/A-bodies	Protein storage in response to stress	Audas et al. (2016), Wang et al. (2018)
15	Cytoplasm	Ubiquitous	P body/RNA processing bodies/GW bodies/decapping bodies	mRNA degradation and silencing	Decker and Parker (2012)
16		Ubiquitous	Pericentriolar matrix	Microtubule nucleation	Mahen and Venkitaraman (2012), Woodruff et al. (2017)
17		Ubiquitous	TIS granule	Helps in 3' UTR-mediated protein-protein interactions	Ma and Mayr (2018)
18		NA*	cGAS condensates	Immune signaling pathway	Du and Chen (2018)
19		Condition-dependent	Stress granule	Regulation of transcription, Storage of RNA in response to stress	Decker and Parker (2012); van Leeuwen and Rabouille (2019)
20		Condition-dependent	Sec bodies	Storage	Zacharogianni et al. (2014), Aguilera-Gomez et al. (2016)
21		Condition-dependent	U-bodies/Uridine-rich snRNP bodies	Storage and assembly of snRNPs	Liu and Gall (2007), Tsalikis et al. (2015)
22		Condition-dependent	Viral factories/Viroplasm/Virus inclusions	Replication and assembly of virus	Heinrich et al. (2018), Alenquer et al. (2019)
23		Cell-type specific (Germ cells)	Balbiani body	Storage	Boke et al. (2016), So et al. (2021)
24		Cell-type specific (Germ cells)	P-granules/Germ granule/polar granule/Chromatoid bodies	Storage	Brangwynne et al. (2009), Marnik and Updike (2019)
25	Membrane-associated	Ubiquitous	Nuclear pore complex	Facilitate selective export/import in nucleus	Schmidt and Gorlich (2016)
26		NA*	ZO-mediated tight junction	Assembly of tight junction	Beutel et al. (2019)
27		Condition-dependent	TCR microcluster	T-cell mediated signal transduction	Su et al. (2016)
28		Condition-dependent	Nephrin cluster	Glomerular filtration barrier	Jones et al. (2006), Banjade and Rosen (2014)
29		Cell-type specific	Synaptic density	Neurotransmission	Zeng et al. (2016), Zeng et al. (2018)

* NA-information not available

## Physio-chemical nature of condensates

Numerous studies using *in vitro* and *in vivo* approaches have provided a solid foundation for understanding the structural and functional aspects of condensates. LLPS can be described as “a thermodynamically driven, reversible, de-mixing process wherein, above a threshold macromolecule concentration, components separate into coexisting dense and dilute liquid phases with different solute concentrations” (Boeynaems et al., 2018; Lyon et al., 2021).

How does the system allow the formation of de-mixed phases? Let us think about the behavior of the multiple copies of two different proteins (X, Y) in a solution. If the interaction between X and Y is energetically more favorable than that between X and X or Y and Y molecules, then the solution will be homogenously mixed, and the system’s entropy will be maximum. However, suppose X and X molecular interaction is energetically favorable over X and Y interaction, in that case, X will get de-mixed from neighboring Y molecules after reaching a critical threshold concentration. Entropy, which generally favors the homogenous distribution of X and Y molecules, gets counteracted by energetically favorable interactions between X and X molecules. The living system also undergoes similar changes where interactions between different molecules lower the energy, driving them to get phase-separated (Mitrea et al., 2018; Hyman et al., 2014; Bhat et al., 2021).

**FIGURE 1:**
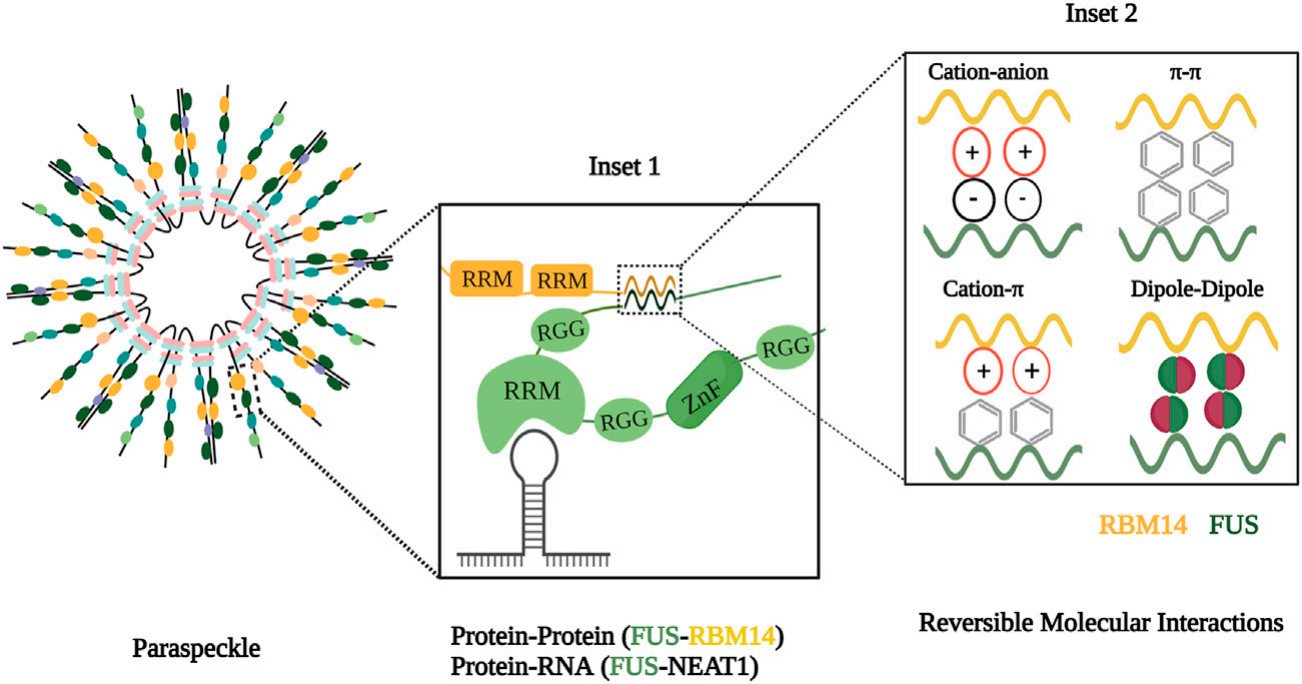
Molecular components and forces involved in condensate formation. Inset 1 illustrates the assembly of paraspeckle comprising scaffolds and clients and interactions between these components. Inset 2 depicts molecular forces with in inter and intramolecular interactions with in phase condensate.

These interactions between molecules such as X and Y can be of both high and low affinity. Most of the high-affinity interactions provide specificity compared to the low-affinity interactions, but multiple weak, cooperative interactions at a higher concentration can also help in the formation of the condensate. These high- and low-affinity interactions include intramolecular and intermolecular interactions such as *π*–*π*, *π*–cation, cation–anion, dipole–dipole, hydrogen–hydrogen, and hydrophobic forces that influence the spatiotemporal arrangement of the molecules in condensates (Figure 1) (Boeynaems et al., 2018; Dignon et al., 2020).

Pioneer studies show that P granules and the nucleolus have liquid-like properties typical of phase condensates. These condensates are 1) spherical, due to interfacial tension between two phases; 2) can undergo fission and fusion processes; 3) have diffuse nature, thereby allowing molecular exchange from the nearby environment to support biological function; and 4) can wet other surfaces upon contact (Brangwynne et al., 2009; Brangwynne et al., 2011; Shin and Brangwynne, 2017).

## Composition of condensates

In a living cell, forces that favor phase separation are mediated by the cell’s biopolymers, such as proteins and nucleic acids. Condensate components such as protein and RNA can be of two types: scaffolds and clients. Scaffold molecules have higher valency, thereby acting as drivers of phase separation, providing a platform for other proteins. These molecules, abundant in condensates, decide the condensate threshold, and their removal likely prevents condensate assembly. Client molecules are recruited for condensation based on their relative stoichiometry with the scaffold. They perform their function by interacting with scaffolds and other client molecules and are generally dispensable for phase separation. For example, in paraspeckles, a nuclear phase condensate, NEAT1 (nuclear-enriched autosomal transcript 1) lncRNA acts as a scaffold, while the RNA binding protein, FUS (fused in sarcoma), is a client protein (Figure 1) (Banani et al., 2017; Dignon et al., 2020). The central feature of the phase-separated condensate is the presence of multivalent molecules to orchestrate intra- or inter-molecular interactions necessary for phase separation. The multivalency in condensates can be achieved by intrinsically disordered regions (IDRs), RNA-binding domains, and posttranslational modifications (Wiedner and Giudice, 2021).

### Protein features for phase separation

Two important features that govern phase separation are the presence of IDR and modular domains. The IDR is a region of amino acids that exhibit low-sequence complexity, are present in a heterogeneous ensemble of conformations, and do not fold into a well-defined three-dimensional structure. They have a higher polar and charged amino acid ratio, including glycine, serine, glutamine, proline, glutamic acid, lysine, and arginine. Even though they have low hydrophobic amino acid content, aromatic amino acids such as tyrosine and phenylalanine are generally interspersed in these regions. These characteristics fulfill the multivalent nature required for phase separation. To phase separate, IDRs can interact with other IDRs of the same protein and/or other proteins. For example, in paraspeckles, RBM14 (RNA binding motif 14) and FUS interact via their IDRs, namely, prion-like domain (Figure 1). These IDRs are present in many proteins such as FUS, YAP (yes-associated protein), and NICD (nephrin intracellular domain) (Banani et al., 2017; Boeynaems et al., 2018; Martin and Holehouse, 2020). Modular domains exhibit discrete secondary and tertiary protein conformations and can interact with other molecules based on their sequence and structure specificity. These domains include RNA recognition motif (RRM), arginine–glycine–glycine motif (RGG), and zinc finger domain. For example, FUS and RBM14 have RRMs, which help them interact with NEAT1 (Figure 1).

### RNA features for phase separation

Studies on phase separation reveal the presence and importance of RNA in condensates. RNA can interact with other RNAs and proteins by virtue of its flexible and variable properties. 1) The most fundamental property imparting multivalency is the negative charge of the RNA backbone through which it can mediate electrostatic interactions with other molecules. Also, the single-stranded nature of RNA exposes its bases and phosphate group to interact with multiple positively charged amino acids or molecules. 2) Flexibility also favors attaining multiple confirmations such as G-quadruplexes, hairpin loops, and helix motifs which provide protein or RNA binding sites. RBPs containing RRMs can recognize these structures. 3) Longer lengths of RNAs like long non-coding RNAs (Figure 1) can provide scaffolding property to the condensate as they can offer multiple binding sites. RNA–RNA interactions are important for mediating phase separation, like in the case of paraspeckle and stress granule formation. In addition to promoting phase separation, excessive or high-affinity RNAs can also prevent phase separation. This inhibition can be due to charge repulsion or blocking protein–protein interactions. RNA can regulate condensate assembly by regulating its dynamic nature or acting as a buffer to prevent abnormal aggregations (Roden and Gladfelter, 2021; Su et al., 2021). For example, RNA can prevent the aggregate formation of FUS in the nucleus since it is present in higher concentrations (Maharana et al., 2018).

## Regulation of phase separation

Apart from the concentration and multivalency of biopolymers, environmental factors such as pH, temperature, hypoxia, and other stress factors can affect the process of condensation. It can also be regulated by different parameters such as posttranslational modifications (PTMs), the presence of a membrane, molecular chaperones, and other active processes. PTMs affect the physio-chemical nature of amino acids, thereby affecting the strength and multivalency of molecules. Membrane surfaces affect the concentration of molecules by either hampering diffusion or promoting transport or crowding of condensate molecules. Similar to PTMs of proteins, modifications of RNA bases alter essential aspects of RNA functions. These posttranscriptional modifications, such as N^6^-methyladenosine modification, can also impact condensate assembly. These modifications can influence phase separation in multiple ways. 1) The modifications give the potential for dynamic and adaptive nature to the RNA. 2) It affects the sequence information and has a substantial impact on the structure of RNA molecules. 3) RNA modifications also affect the processing of RNAs. These changes altogether mediate adaptive RNA–RNA and RNA–protein interactions. These factors cumulatively make condensation an adaptive process and only happen upon receiving specific cues from the environment nearby. Thus, the relative stoichiometry of condensate components controls their dwell time within condensate and consequently fine-tunes functions performed by them. (Darino and Schaefer, 2018; Snead and Gladfelter, 2019; Lu et al., 2021; Riback et al., 2017; Roden and Gladfelter, 2021; and Wiedner and Giudice, 2021).

## LncRNA, “the anchor” of phase separation

LncRNAs are emerging as important players in the formation of biomolecular condensates. Other RNAs such as mRNAs, rRNAs, and tRNAs have been shown to play important roles in various condensates such as stress granules and P bodies (Protter and Parker, 2016), but decoding the essentiality of lncRNA in phase separation is in its initial stage. LncRNAs do not perform an enzymatic function like a protein nor do they code for a polypeptide like an mRNA, but they have the ability to bring the components together required for a particular biological function, holding them firmly and compartmentalizing them in their contour of action where they can perform their tasks more efficiently. This potential of lncRNAs makes them emerge as anchors of phase separation.

Several features within these anchors have been shown to aid them in organizing a phase condensate. One of these features is their length. LncRNAs being longer than other non-coding RNAs such as miRNA can provide a larger platform for binding RBPs (RNA-binding proteins), as with NORAD lncRNA that has 18 Pumilio binding motifs (Elguindy and Mendell, 2021), providing them better sequestration capacity. They do not contain a specific domain similar to IDRs (Wright and Dyson, 2015) present in proteins forming condensates, but their ability to bind RBPs having IDRs can assist in undergoing phase separation. For example, HNRNPA1 in HSATIII lncRNA nuclear stress bodies has an IDR that mediates protein–protein interaction between numerous HNRNPA1 molecules to cause phase separation (Aly et al., 2019).

The presence of lncRNAs within a condensate makes it less viscous, thus giving a more liquid-like nature compared to the increased protein component, which imparts a solid nature to the condensate (Elbaum-Garfinckle et al., 2015). This protein-to-RNA ratio increase may also prevent condensate formation (Maharana et al., 2018). The fluidity generated in the condensate due to the presence of lncRNAs makes it more dynamic, enhances the rate of molecular exchange, and eases the assembly and disassembly process. Studies on several biomolecular condensates suggest poorly translated mRNAs favor phase separation (Khong et al., 2017). Aptly, lncRNAs, known for their inability to code, hint at their essential role in phase separation.

For any protein or RNA, reaching a threshold concentration for phase separation is necessary (Banani et al., 2017). Therefore, the lncRNAs that form part of condensates are generally upregulated, which may be associated with a cancerous condition such as TNBL (tumor-associated NBL2 transcript) upregulated in colorectal cancer (Dumbovic et al., 2018) or during normal processes such as DIGIT upregulated during endoderm differentiation (Dhaneshvar et al., 2020).

In addition, several reports suggest that inhibiting lncRNAs either by knockdown, antisense oligonucleotides, RNases, RNA pol II inhibitors, or deleting their protein-interacting regions hinders condensate formation, thereby negatively affecting the normal function of the condensate and its components (Pessina et al., 2019; Huo et al., 2020; Lee et al., 2021). This again points toward the importance of lncRNAs in biomolecular condensates and suggests they are the anchors of phase separation. Various other features of lncRNAs may facilitate the compartmentalization process, such as sequence or nucleotide composition, length, secondary structures, and modifications. Some of the important features associated with lncRNAs that form condensates like their subcellular localization, their interacting partners, and the biological functions they assist in are summarized (Table 2).

**TABLE 2:** LncRNAs that are associated with phase condensates.

S. No.	LncRNAs	Subcellular localization	Interacting proteins	Process affected	Biological function	Salient features	References
1	*DIGIT*	Nuclear	BRD3, SMARCD1	Transcription	Endoderm differentiation	Interaction with histone reader domain	Dhaneshwar et al. (2020)
2	*dilncRNA*	Nuclear	53BP1	DNA repair	DNA damage response	Processing into shorter RNAs	Pessina et al. (2019)
3	*eRNA*	Nuclear	YTHDC1	Transcription	Proinflammatory gene expression	Interaction with histone reader domain; m^6^A modification.	Lee et al. (2021)
4	*HSATII*	Nuclear	MeCP2	Transcription	Cancer associated sequestration of chromatin regulatory proteins	Repetitive sequences	Hall et al. (2017)
5	*HSATIII*	Nuclear	HNRNPA1, HNRNPH1, HNRNPM	Pre-mRNA processing	Sequestration in response to stress	Repetitive sequences	Aly et al. (2019)
6	hsrω-n	Nuclear	HNRNPs—HRB87F, Hrp40, Hrb57a, S5	Pre-mRNA processing	Prevent promiscuous RNA processing in response to heat shock	Repetitive sequence	Prasanth et al. (2000)
7	MajSat	Nuclear	SAFB	Chromatin remodelling	Heterochromatin stabilization	Repetitive sequence	Huo et al. (2020)
8	*meiRNA*	Nuclear	Mei2p, Mmi1	Transcription and pre-mRNA processing	Decoy Mmi1 to promote meiosis	Two isoforms; hexanucleotide repeats in 3' region	Shihcino et al. (2015)
9	NEAT1	Nuclear	NONO, SFPQ, RBM14, FUS, HNRNPH3	Pre-mRNA splicing, mRNA nuclear retention	Role in specific tissue development and cancer	Two isoforms; 3' triple helix instead of poly-A tail	Clemson et al. (2009), Hirose et al. (2019)
10	*NORAD*	Nuclear	PUM1/2	Genomic stability	Sequester destabilizing proteins PUM1/2	Repeated PUM binding motifs	Elguindy et al. (2021)
11	*PNCTR*	Nuclear	PTBP1	Splicing	Control Alternate splicing and promote cell survival	Short Tandem repeats	Yap et al. (2018)
12	*SNGH9*	Cytoplasmic	Phosphatidic acid, LATS1	Cell signalling	Promote oncogenic YAP signalling	Association with a lipid	Li et al. (2021)
13	*TERRA*	Nuclear	RAD52, BLM	DNA repair	Alternative lengthening of telomeres in cancer	Form RNA-DNA hybrid and acts as a template	Min et al. (2019)
14	*TNBL*	Nuclear	NPM1, SAM68, CELF1	Genome organisation, splicing and mRNA stability	Sequestration of proteins in response to cancer.	Repetitive sequence	Dumbovic et al. (2018)
15	*Xist*	Nuclear	PTBP1, MATR3, TDP43, CELF1	Transcription	X chromosome inactivation	Repetitive sequences	Pandya-Jones et al. (2020)

**FIGURE 2:**
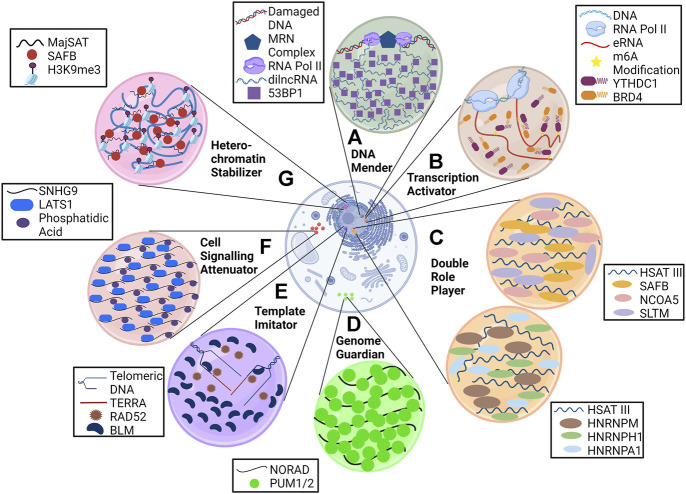
LncRNAs are anchors of phase condensates. LncRNAs orchestrate the formation of phase condensates and execute various processes such as **(A)** DNA repair (dilncRNA), **(B)** transcription activation (eRNA), **(C)** pre-mRNA splicing and transcription regulation (HSATIII), **(D)** genome stabilization (NORAD), **(E)** alternative lengthening of telomeres (TERRA), and **(F)** cell signaling (SNHG9).

Some of the lncRNAs reported to be the mainstay in phase separation (Figure 2) are briefly discussed in the sub-sections below.

### The heterochromatin stabilizer

MajSAT lncRNA is transcribed from heterochromatin-abundant repetitive elements with which a nuclear matrix (NM)–associated protein, SAFB (scaffold attachment factor B), interacts via its R-/G-rich region. This association forms foci in DAPI-dense regions of the nucleus (heterochromatin region), where SAFB mediates pericentromeric heterochromatin (PCH) stabilization. Downregulation of MajSAT lncRNA with antisense oligonucleotides leads to dissociation of the SAFB condensate, thereby causing weakening of PCH foci as indicated by H3K9me3 staining (Huo et al., 2020).

### The transcription activator

Enhancer RNAs (eRNAs), as the name suggests, are long non-coding RNA transcribed from enhancer regions and have a crucial role in transcriptional regulation. They have a heavy deposition of m^6^A (N^6^-methyladenosine) modification, as characterized by methylation-inscribed nascent transcript sequencing (MINT-seq) (Lee et al., 2021). These modified eRNAs mark the enhancer regions for the recruitment of m^6^A reader YTHDC1, forming a phase condensate that facilitates the formation of the BRD4 coactivator condensate, causing transcription activation. Knocking down eRNA or METTL3 and METTL14 (leading to the removal of m^6^A marks) causes a reduction in YTHDC1 binding to the enhancer, thereby reducing the gene expression. *In vitro* analysis of YTHDC1/m^6^A-eRNA suggested that YTHDC1 cannot form the condensate alone.

### The cell signaling attenuator

The role of phase condensates in cell signaling is beginning to be deciphered. SNHG9 (small nucleolar RNA host gene 9) lncRNA is now known to regulate the Hippo pathway (Li et al., 2021) by physically interacting with phosphatidic acid (PA) and LATS1 (large tumor suppressor kinase 1) to form a puncta. SNHG9 within this puncta promotes PA-mediated inactivation of LATS1, which causes increased YAP (yes-associated protein) activation and nuclear localization, leading to cancer progression. Knocking down SNHG9 or deleting its PA-interacting region leads to reduced LATS1 puncta formation and sequestration of YAP in the cytoplasm.

### The genome guardian

NORAD (non-coding RNA activated by DNA damage) lncRNA is localized in the cytoplasm and sequesters Pumilio proteins (PUM1 and PUM2) in a condensate. Pumilio proteins are RNA-binding proteins that are posttranscriptional repressors and cause translation inhibition and degradation of several mRNAs that include important regulators of mitosis. Negative regulation of PUM proteins by NORAD prevents abnormal mitosis and maintains genomic stability in mammalian cells. NORAD is a perfect example of RNA imparting multivalency in biomolecular condensate as it has 18 PUM response elements (PRE) where PUM proteins bind. A decrease in PREs leads to reduced phase separating ability and requires higher PUM concentration to form condensates (Elguindy and Mendell, 2021). Knockdown of NORAD leads to diffused localization of PUM proteins, significant repression of PUM targets, and higher chromosomal segregation errors.

### The template imitator

LncRNAs such as TERRA (telomeric repeat-containing RNA) are known to be a part of ALT-associated pro-myelocytic leukemia bodies (APBs) clustered on the telomeres. Along with TERRA, other DNA replication and repair proteins such as BLM (helicase) and RAD52 (RNA-templated DNA repair) are present in APBs. These bodies mediate telomerase-independent telomere maintenance known as alternative lengthening of telomeres (ALT) in cancer cells, where TERRA acts as a template for telomeric DNA synthesis. Knockdown of associated proteins leads to a reduction in APBs (Min et al., 2019), but the effect of TERRA depletion on it is not known. However, it is known that loss of TERRA results in telomere dysfunction and instability (Chu et al., 2017). Higher expression of TERRA in ALT cells (Episkopou et al., 2014) may have a role in phase separation, but this needs to be further elucidated.

### The DNA mender

Double-strand breaks (DSBs) in DNA are sensed by the MRE11-RAD50-NBS1 (MRN) complex, which recruits RNA polymerase II at the damaged sites. This recruitment leads to the synthesis of dilncRNAs (damage-induced long non-coding RNAs) (Sharma et al., 2021), which are further processed into shorter DDR (DNA damage response) RNA. These RNAs form a DNA–RNA hybrid with damaged ends favoring repair by homologous recombination and causing accumulation of DDR factors, such as 53BP1, into foci. Treatment with RNAase A, RNA pol II inhibitors, or antisense oligonucleotides against dilncRNA inhibits this foci formation and even disrupts already formed foci. This, in turn, causes reduced DNA repair efficacy (Pessina et al., 2019).

### The double role player

As mentioned earlier, lncRNAs provide binding platforms for various RBPs, but it is not necessary that a particular lncRNA interacts with only a single type of RBP. Various features of lncRNA, especially the repetitive nature, give them the liberty to bind multiple RBPs. One such versatile long non-coding RNA is HSAT III (highly repetitive satellite III) which is now known to provide architecture to two distinct types of nuclear stress bodies under thermal stress. First is nSB-S, in which SAFB associates with HSAT III for the transcription of heat shock proteins. The second is nSB-M, where HNRNPM (heterogeneous nuclear ribonucleoprotein M) interacts with HSAT III and affects pre-mRNA splicing. Knockdown of HSAT III with antisense oligonucleotides causes the disappearance of both types of stress bodies (Aly et al., 2019).

## Importance of phase separation in cancer

Over the years, people have been trying to understand the role of cancer driver mutations and their role in cancer progression. Interestingly, it was found that specific mutation that leads to oncogene activation also results in the formation of super-enhancers, which is a type of phase condensates that are formed by a clustered DNA element (Bradner et al., 2017; Cho et al., 2018). Many reports documented the involvement of phase condensation in regulating several functions of cancer cells (Boija et al., 2021). However, further investigation is needed on the mechanistic detail of phase separation involvement in cancer development and progression, which will help improve the therapeutic strategies.

## Cancer-associated LncRNAs in phase separation

Long-noncoding RNAs are dysregulated in cancer and have an important role in cancer development and progression. It is challenging to completely understand lncRNA’s function due to its large size, conformational flexibility, and low abundance. However, the specific subcellular localization of lncRNA leads to stoichiometrically measurable effects (Wu et al., 2021). LncRNA has the potential to induce phase separation, but the role of lncRNA on phase separation for cancer development and progression is now at the stage of its infancy.

The NEAT1 lncRNA, a dysregulated lncRNA in cancer, is an architectural component of the paraspeckle nuclear body. In the presence of DNA damage response (DDR), p53 protein is activated, which leads to the NEAT1-mediated paraspeckle formation that attenuates oncogene-dependent activation of p53 (Adriaens et al., 2016). MALAT1 (metastasis-associated lung adenocarcinoma transcript 1) is known to form nuclear speckles comprising pre-mRNA splicing and transcription factors (Hutchinson et al., 2007; Miyagawa et al., 2012). Moreover, MALAT1 has an essential role in metastasis and patient survival in non–small cell lung carcinoma (Ji et al., 2003). Interestingly, another report shows that MALAT1 translocates to a heat-inducible non-coding RNA containing a nuclear body (HiNoCo body) from nuclear speckles in response to heat shock in a heat shock factor 1–independent manner (Onoguchi-Mizutani et al., 2021). They have also shown that the HiNoCo body is a reversible phase condensate and MALAT1 is essential for cell proliferation under heat shock. It has been shown that Hippo signaling pathway is a tumor suppressor pathway by attenuating the YAP nuclear translocation, and most cancer cells are characterized by dysregulation of the Hippo pathway (Pan, 2010; Zhou et al., 2015). However, the lncRNA association with the Hippo signaling pathway is largely unknown. Recently, Li et al., 2006 showed that SNHG9 (small nucleolar RNA host gene 9), a lipid-associated tumor-promoting lncRNA, and phosphatidic acid (PA) facilitate attenuation of LATS1 kinase activity by inducing LLPS, which sequesters LATS1 to promote oncogenic YAP signaling (Li et al., 2021).

Glutamine metabolism is an important source of carbon and nitrogen for several biosynthetic processes. The rate-limiting step of glutaminolysis is the generation of glutamate from glutamine catalyzed by glutaminase-1 (GLS1) (Yoo et al., 2020). Glutamine is the critical nutrient source for many cancer cells (DeBerardinis and Cheng., 2010). The lncRNA GIRGL (glutamine insufficiency regulator of glutaminase lncRNA), which is induced upon glutamine starvation, causes dimerization of CAPRIN1. This complex interacts with GLS1 mRNA, resulting in the formation of phase condensate facilitated by LLPS. In this process, CAPRIN1 suppresses GLS1 mRNA translation which influences cancer cell survival under prolonged glutamine deprivation stress (Wang R. et al., 2021).

## Conclusion and future directions

Phase separation provides a framework to understand how an individual cell coordinates specific processes at subcellular levels that are simultaneously robust and adaptive. Like the membranous barrier, phase boundary ensures the controlled localization of biomolecules without the complication of transport through a barrier. The formation of biological condensates is a tightly regulated thermodynamic process that depends on the abundance, sequence, and structure of scaffold and client molecules. Various key mechanisms such as environmental conditions, chaperones, presence of a membrane, and posttranslational modifications can work in concert to affect the physio-chemical property, function, and homeostasis of condensates.

LncRNAs are key molecules in a cell, whether they are part of the phase condensate or not, as they perform various functions in the cell such as gene regulation, including genetic and epigenetic modifications, swimming to different locations within the cell, and having the potential to interact with several biopolymers such as DNA, RNA, proteins, and lipids. The knowledge of LLPS and of lncRNAs being part of phase condensates unfolds whole new possibilities. This efflorescing concept gives a better understanding of how lncRNAs can get concentrated within a particular cell site while interacting with various biopolymers and performing specialized tasks. Further exploration of phase condensation may reveal secrets about the additional functions of known lncRNAs. LLPS can, indeed, be an educator, answering various unsolved questions and providing space to lncRNAs to execute their superpowers. However, a few questions remain to be clarified, like how a few condensates of a particular lncRNA can define its activity at thousands or more locations? Are various effector sites part of a single condensate? While the dynamic nature of phase condensates is well-appreciated, a better understanding of whether this mobility can help simultaneously reach the enormous number of effector sites is needed.

Though phase separation is important for normal cell functioning, some phase-separated condensates are only present in cancer cells, such as FUS-CHOP-mediated phase separation is present in sarcoma (Pérez-Losada et al., 2000). Some condensates can be present in both normal and transformed cells, but the higher expression of proteins involved in phase separation in cancer cells can promote oncogenic signaling. Since phase separation is adaptive, it can respond to oncogenic alterations such as mutations, localization, and degradation of molecules, which regulate the phase separation process either positively or negatively (Gao et al., 2019; Wang B. et al., 2021). Based on the involvement of phase separation in different cancer-related biological processes, altering the expression of critical components through phase separation can be used for targeted therapy. These molecules can include scaffold and client molecules and the environmental conditions involved in phase separation.

The studies on lncRNA-associated liquid–liquid phase separation in cancer are limited. Further investigation is required to improve the understanding of the role of lncRNA for LLPS formation in cancer to improve the therapeutic strategies. In addition, phase condensates may increase the drug efficacy or drug resistance in cancer cells depending on their physicochemical properties to form phase condensates (Klein et al., 2020). It can be hypothesized that phase separation sequesters drugs given to eliminate the cancer cells and lncRNAs may be playing an essential role in it, thereby altering drug efficacy. Where lncRNA is required for the phase condensate, strategies to target lncRNA could be considered. In cases where lncRNA is just a part of phase condensate but is not essential for the architecture itself, methods to inhibit the phase condensate formation may be beneficial. Furthermore, targeting phase separation may form an important therapeutic strategy in the future, especially for undruggable proteins for their intrinsically disordered nature, which is highly abundant in eukaryotes.

## Abbreviations

CAPRIN1, Cytoplasmic Activation- And Proliferation-Associated Protein 1; DDR, DNA damage response; GLS1, glutaminase-1; GIRGL, glutamine insufficiency regulator of glutaminase lncRNA; HNRNPA1, heterogeneous nuclear ribonucleoprotein A1; HSATIII, highly repetitive satellite III; LATS1, large tumor suppressor kinase 1; LLPS, Liquid-Liquid Phase Separation; MALAT1, metastasis associated lung adenocarcinoma transcript 1; MINT-seq, methylation-inscribed nascent transcripts sequencing; NICD—Nephrin intracellular domain, NORAD, non-coding RNA activated by DNA damage; NEAT1, Nuclear enriched autosomal transcript 1; FUS, Fused in sarcoma; IDRs, Intrinsically disordered regions; PA, phosphatidic acid; PRE, PUM response elements; PTMs, posttranslational modifications; RBM14, RNA binding motif protein 14; SAFB, scaffold attachment factor B; SNHG9, small nucleolar RNA host gene 9; TERRA, telomeric repeat-containing RNA; TNBL, tumor-associated NBL2 transcript; YAP, Yes-associated protein.
